# Current Status and Major Challenges to the Safety and Efficacy Presented by Chinese Herbal Medicine

**DOI:** 10.3390/medicines6010014

**Published:** 2019-01-18

**Authors:** Xian Zhou, Chun-Guang Li, Dennis Chang, Alan Bensoussan

**Affiliations:** NICM Health Research Institute, Western Sydney University, Penrith, NSW 2751, Australia; c.li@westernsydney.edu.au (C.-G.L.); d.chang@westernsydney.edu.au (D.C.); a.bensoussan@westernsydney.edu.au (A.B.)

**Keywords:** Traditional Chinese medicine, Chinese herbal medicine, safety, efficacy, herb–drug interaction, synergy, herbal formulae

## Abstract

Traditional Chinese medicine (TCM) is not only used prevalently in Asian countries but has also gained a stable market globally. As a principal form of TCM, Chinese herbal medicine (CHM) is comprised of treatments using multiple Chinese herbs which have complex chemical profiles. Due to a lack of understanding of its modality and a lack of standardization, there are significant challenges associated with regulating CHM’s safety for practice and understanding its mechanisms of efficacy. Currently, there are many issues that need to be overcome in regard to the safety and efficacy of CHM for the further development of evidence-based practices. There is a need to better understand the mechanisms behind the efficacy of CHM, and develop proper quality standards and regulations to ensure a similar safety standard as Western drugs. This paper outlines the status of CHM in terms of its safety and efficacy and attempts to provide approaches to address these issues.

## 1. Introduction

Traditional Chinese medicine (TCM) is an integrated system of primary healthcare that originated in ancient China and evolved over 5000 years. It is one of the oldest and most long-standing healthcare systems in the world and was rooted in the ancient Chinese philosophy of Taoism. It is prevalent throughout east and south-east Asian countries, including Japan, Korea and Vietnam. TCM encompasses many different practices, including Chinese herbal medicine (CHM), acupuncture, moxibustion, Tui Na, dietary therapy, Tai Chi and Qi Gong. Notwithstanding this diversity, CHM is the mainstay and principal form of TCM practice [1].

CHM comprises a pharmacopoeia of thousands of medicinal substances, called Chinese Materia Medica (CMM), primarily plants and some minerals and animal substances [2]. In clinical practice, 200 to 600 CMM are routinely utilised by Chinese medicine practitioners, with 50 considered as ‘fundamental’ [3]. CHM is increasingly in great demand not only in Asian countries but also in many western countries, with an international market estimated at approximately US$1.025 billion in 2016 [4]. Depending on different definitions and calculations, CHM represents about 20–50% of the herbal medicine market share worldwide [5]. In Western countries, CHM has been recognized as a popular form of complementary and alternative medicine with the belief that CHM incurs fewer side effects, as it is generally extracted from natural products without artificial additives [6,7].

Although TCM has been clinically practised for thousands of years, most CHM products do not possess up-to-date data regarding their safety and modern scientific evidence for their claimed clinical uses. This shortcoming of CHM restricts its ongoing development and its acceptance and promotion by mainstream healthcare systems internationally. Given the fact that the philosophy, diagnosis, prescription principles and processing methods of CHM remedies are completely different to modern Western Medicine (WM), attempts to manage and regulate it in a scientific and modernised manner have faced great challenges. Today, there are still numerous issues associated with the safety of CHM products, including product quality, improper practice, drug/herb–herb interactions, contaminants and toxicities. Additionally, the mechanisms of its efficacy remain largely unclear. The World Health Organization (WHO) listed the main challenges for the efficacy issues related to CHM products, including a lack of research data, and a lack of appropriate mechanisms to control and regulate advertising, claimed clinical uses and herbal products [8]. With a steadily growing industry and the popular use of CHM worldwide, greater effort should be directed towards its safety and efficacy in order to minimise the safety risks and provide scientific proof and clinical evaluation for the continuous development of this ancient medical system [9].

This review provides a broad view of CHM, specifically focusing on current issues regarding its safety and efficacy. The complexity of these issues, possible solutions and future directions are also discussed.

## 2. Challenges to the Safety of CHM

Generally speaking, CHM is relatively safe compared to WM, as demonstrated from overall adverse reactions reported in literature, although CHM must conform to modern safety requirements in order to fulfil its role as a contemporary treatment option. CHM products are derived from nature, and some can be toxic to humans. The general belief that all CHM products pose lower risks than synthetic drugs is a misconception. In fact, any substances that affect the body’s defence or physiology may have various degrees of side effects. Despite the potential harm from several Chinese medicinal herbs with predictable risks, many safety issues are caused by poor quality controls, inappropriate prescriptions and administration and a lack of safety instructions [10].

### 2.1. Inherent Toxicity

All medicines carry risks which are related to their uses and forms presented. Serious toxicities/adverse reactions to WM are often well-understood and regulated. However, the inherent toxicities for CHM products are generally underestimated, especially when they are used as dietary supplements or use in a long peroid.

A large number of Chinese herbs are known to be toxic to humans. One of the most extensive modern records, the Encyclopaedia of Materia Medica [*Zhong Yao Da Ci Dian*] (1977), documents 495 out of 5767 medicinal herbs as being toxic [11]. For example, aristolochic acid, isolated from *Aristolochia debilis* Sieb. et Zucc. (known as *Madouling* in CHM), can lead to rapid kidney failure, as seen in a group of women who had all taken the same weight-loss supplement [12]. *Glycyrrhiza* spp. (liquorice root) and its extracts, which are often used in CHM, as an expectorant, a corrective adjunct and a harmonising ingredient in many formula preparations [13], contains mineralocorticoid, which can cause adverse reactions such as oedema, hypertension and electrolyte imbalances [14]. *Panax ginseng* C. A. Mey. (known as *Renshen* or *Hongshen* in CHM), one of the most popular CHM herbs and used for a variety of conditions, including tiredness and poor circulation, has been associated with an ‘abuse syndrome’ characterised by insomnia, irritability and excitability [15].

The vast majority of toxic CHM substances cause safety issues that are predictable. Based on thousands of years of accumulated traditional knowledge and clinical practice, the precautions, warnings and contraindications for commonly used forms of CHM (both in terms of individual herbs and formulae) are documented in pharmacopoeias, monographs and databases in Chinese. There are many encyclopaedias and monographs in Chinese (some translations into English are in progress) containing safety information, such as the Pharmacopoeia of the People’s Republic of China (PPRC), Toxic Chinese Materia Medica: Past and Present [*Du Ju Zhong Yao Gu Jin Yong*], Encyclopedia of Toxic Chinese Materia Medica [*You Du Zhong Cao Yao Da Ci Dian*], Toxic Drugs Herbal [*Du Yao Ben Cao*] and Modern Toxicology of Chinese Materia Medica [*Xian Dai Zhong Yao Du Li Xue*] [16]. Modern documentations on CHM toxicity are primarily based on compilations of scientific data generated from isolated or purified chemical ingredients in these herbs. For example, the Australia’s Therapeutic Goods Administration (TGA) established the ‘poison standard’ that consists of decisions regarding the classification of medicines (including toxic compounds and herbal ingredients from CHM) and poisons into Schedules, for inclusion in the relevant legislation of the States and Territories. For example, aconite has been included in Schedule 4 of the Standard of the Uniform Scheduling of Medicines and Poisons (SUSMP), and can only be prescribed by registered medical practitioners, dentists or veterinarians and dispensed by registered pharmacists [17]. In 2015, the Chinese Medicine Board of Australia (CMBA) commissioned the National Institute of Complementary Medicines (NICM Health Research Institute) to produce a new Nomenclature Compendium for CHM, which extracted restrictions and warnings from SUSMP and PPRC for over 600 of the most commonly used CHM, which greatly saves time and efforts for TCM practitioners referring to the safety information [18]. However, due to the nature of CHM practice, which relies largely on direct clinical experience over time, the precise toxicity data for most CHM are still limited, compared to modern pharmaceuticals [16]. Presently, adequate data regarding toxic herbs, toxic target organs, safe dose range, safety window of effective dose, minimum toxic dose and herb–herb/drug interactions are still lacking [19].

### 2.2. Safety Risks in the Practice of CHM

The knowledge and qualification of practitioners have a direct bearing on patient safety [8]. The safety risks identified in CHM practice include misdiagnosis, poor prescribing, failure to observe and/or explain contraindications, inappropriate dosage and duration of therapy, and failure to avoid known interactions with pharmaceuticals and precautions associated with a particular substance [20]. Inappropriate practice can be attributed to two reasons: Lack of regulation and inadequate training/education. According to a WHO report from 129 member states, up to 43.5% of traditional medicine practitioners were not regulated in 2012, and 56% of these practitioners did not have university-level education [8].

To avoid the safety issues caused by inappropriate use and practice, greater attention and efforts have to be made to ensure the education level and to strengthen the regulation of TCM practitioners. It is recommended that general practitioners (GPs) and pharmacists be trained with the safety knowledge of CHM so that patients can be instructed [21]. In China, all TCM practitioners are required to receive education and pass a Chinese medicine degree to be registered and practice legally. All TCM practitioners are regulated under the State Administration of Traditional Chinese Medicine. However, aside from Australia, no Western countries have established a national regulation of TCM practitioners. Some countries such as the United States have regulations on certain TCM therapies such as acupuncture. In 2012, Australia became the only Western country to introduce a national registration for Chinese medicine practitioner. All TCM practitioners must be registered under the national registration and accreditation scheme with the CMBA to practice. They are required to master knowledge of the toxic and restricted herbs to lower the safety risks of prescribing CHM in Australia. As most Western nations have yet to start official regulation for TCM practitioners, the WHO put this concern into their Traditional Medicine Strategy report 2014–2023, as well as relevant strategies and actions to strengthen the safe use of CHM products by practitioners worldwide [8].

### 2.3. Poor Quality Control

Poor quality control of CHM includes adulterated herbal products, misidentification/mislabelling and contamination. These can lead to adverse events.

Instances of CHM being adulterated with pharmaceutical drugs have repeatedly been documented. Pharmaceutical drugs that are commonly used for adulteration include non-steroidal anti-inflammatory agents, sedatives and corticosteroids such as ibuprofen, indomethacin, ketoprofen and mefenamic acid [22]. A systematic review from Ernst (2002) summarised the data of CHM products adulterated with conventional drugs collected from six databases (Medline, Embase, Biosis, Amed, Cochrane Library and CISCOM) and revealed that the adulteration in the CHM products has led to 18 adverse reaction case reports, including two serious adverse events and four cases with contamination detected in their chemical fingerprints, one led to a fatality and at least were six potentially life-threatening [23].

Misidentification and mislabelling due to poor quality manufacturing can lead to adverse events. The California Department of Health Services investigated a poisoning case in which the herbal ingredient was found to be *Podophyllum emodi* Wall. var. *chinense* Sprague (known as *Taoerqi* in CHM) which contained podophyllotoxin rather than the supposed *Clematis chinensis* Osbeck (known as *Weilingxian* in CHM). The misidentification led to permanent peripheral neuropathy in the patient [24].

Incorrect nomenclature in the labelling of CHM products can raise potential safety risks for consumers. If a CHM product is labelled with the common English name only, it can be difficult to trace its species, pharmaceutical names and botanical name. For example, ginseng products should specify the pharmaceutical name as Ginseng Radix et Rhizoma Rubra (*Panax ginseng* C. A. Mey., Korean Ginseng, known as *Hongshen* in CHM), or Panacis Quinquefolii Radix (*Panax quinquefolium* L., American Ginseng, known as *Xiyangshen* in CHM) or other ginsengs [25]. Incorrect dosage can also cause severe safety problems. At least a dozen deaths, heart attacks and strokes have been reported due to the over-dosage of ephedra (known as *Mahuang* in CHM) [26,27].

Failure to detect and regulate excessive contamination in CHM products is a health hazard. Posadzki et al. (2013) published an overview of 26 high-quality systematic reviews that summarised and critically evaluated the evidence of the contamination of CHM. It reported that, most commonly, CHM products were contaminated with dust, pollens, insects, rodents, parasites, microbes, fungi, mould, toxins, pesticides and/or toxic heavy metals [28]. The most severe adverse effects caused by these contaminations include agranulocytosis, meningitis, multi-organ failure, perinatal stroke, arsenic, lead or mercury poisoning, malignancies or carcinomas, hepatic encephalopathy, hepatorenal syndrome, nephrotoxicity, rhabdomyolysis, metabolic acidosis, renal or liver failure, cerebral oedema, coma, intracerebral haemorrhage and death [28]. In 2015, a group of researchers from Curtin University, Murdoch University and the University of Adelaide conducted genetic analysis to assess the molecular content of 26 widely available CHM products, and reported that 90% were not fit for human consumption. Their results showed that more than 90% of the tested products were detected to have some form of contamination and/or substitution. Further, mass spectrometry revealed heavy metals including arsenic, lead and cadmium, one with a level of arsenic >10 times the acceptable limit and beyond the safe ingestion recommendations [29].

Safety issues caused by contamination, heavy metal and adulteration in the CHM products are mainly attributed to the limited quality control regulations and lack of internationally accepted quality standard of CHM. The WHO guidelines on good agricultural and collection practices (GCAP) for medicinal plants contribute to the quality control for both the raw herbal medicines and finished herbal products, although it is not specifically for CHM. In these guidelines, information regarding good agricultural practices including identification/authentification, seeds, cultivation, harvest and personnel is provided [30]. The international Organization for Standardization (ISO) is currently developing a range of quality standards of CHM, including raw materials and finished manufacture products, which may help develop and regulate the international market for CHM products.

Different regulations on CHM in different countries may affect CHM associated risks. Countries like China, Korea and Japan all have a well-established regulatory system, which is based on traditional practice and national standards on CHM. Many western countries, on the other hand, often follow regulations for foods, food supplements or pharmaceutical products, without special considerations of CHM in general. The effectiveness of these regulations in minimising the CHM associated risks is yet to be established. In Australia, for example, Good Manufacturing Practice (GMP) has provided a framework to ensure the quality and ability to deal appropriately and quickly with problems when they do arise. Each Australian manufacturer of listed herbal medicine products must hold a manufacturing licence and comply with the Australian Code of GMP for Medicinal Products regarding manufacturing, packaging and labelling [10]. The herbal ingredients/chemical substances contained in the formula or CHM products that may cause potential toxicity to humans should follow the regulation. Some environmental related factors can be controlled by implementing standard operating procedures (SOP) including GAP, good laboratory practice (GLP), good supply practice (GSP) and GMP.

However, the potential safety risks of raw herbal materials need a particular attention as currently there is lack of regulations. As most of the herbal materials for CHM products in Western nations are imported from China, the examination of restricted substances and endangered species by Quarantine and Customs is evidently insufficient for medicinal purposes. There is an urgent need to establish a national regulation and quality testing system for raw herb materials and their extract preparations. To avoid potential safety issues, proper quality control processes for identification and quantification based on monographs and/or pharmacopoeias should be conducted, including examination of appearance, microscopic/macroscopic analysis and chemical fingerprinting. Chemical approaches (i.e., mass spectrometry, DNA fingerprinting) that would detect any contamination, and the presence of pollutants, should be conducted. Any study conducted on whole plants or extracts must use only botanically/chemically authenticated material. It is recommended that the quality control of raw herbs and extracts, which are equally important as the finished products, be considered and regulated officially.

### 2.4. Self-Medication and Drug-Herb Interaction

In most Asian countries, CHM products can be obtained with a practitioner’s prescription as well as through over-the-counter (OTC) self-prescription. During self-prescription, there is no guarantee that safety information is fully available or that customers will follow the safety instructions, which may pose a risk for self-prescribed patients [21]. A number of self-poisoning cases using CHM have been reported. In 2011, a cross-sectional telephone survey of Hong Kong Chinese adults (*n* = 1100), with informed verbal consent, revealed that 71.7% (789/1100) reported using OTC traditional CHM in the past year, and 2.3% (25/1100) reported at least one adverse event during that time. Of the 27 adverse event cases reported among OTC CHM users, the most common adverse events were allergic reactions (*n* = 11) dizziness (*n* = 5) and gastrointestinal problems (*n* = 4) [31].

For self-prescription of OTC CHM products, consumers rely on product labels for safety information. However, limited accessibility of reliable information on product labels has been reported, attributed to the potential risks for self-medicated patients. Moreover, the TCM indications—such as ‘dispelling dampness’ or ‘normalising the gall-bladder’—were difficult for consumers to understand and interpret, as TCM terminology requires professional knowledge of TCM concepts [31]. Another barrier was the perception that reliable knowledge of OTC CHM products could not be obtained from Western medical professionals [31]. Therefore, self-management of health using OTC CHM products with limited accurate and reliable safety information has the potential to lead to inappropriate use and safety issues.

In some cases, patients may use traditional and conventional medicine simultaneously without informing their GP. The likelihood of adverse reactions has been reported through drug–herb interactions [32,33]. For example, *Salvia miltiorrhiza* Bge. (known as *Danshen* in CHM) was reported to exaggerate the anti-coagulant response to warfarin [34]. *Panax ginseng* C. A. Mey. (known as *Renshen* or *Hongshen* in CHM) was also known to interact with warfarin, decreasing the international normalised ratio (INR) [35]. *Ginkgo biloba* L. (known as *Yinxingye* in CHM) was suspected to interact with ibuprofen, leading to fatal intracerebral mass bleeding [36]. Since the mechanism of the interactions is often not clear, it can certainly raise safety concerns for self-medicating if this is done without instructions and not carefully monitored [21].

OTC CHM product-related adverse events are an underappreciated public health issue that may require greater scrutiny and improved surveillance. Improved surveillance of complementary medicines should be prioritised by governments in order to provide more comprehensive safety information for health professionals and consumers. It is a necessity that the information about validated precautions, interactions and side effects are clearly stated with the product and provided to consumers when they purchase herbal products [37]. This is required to strengthen the current regulation and quality control standards for CHM worldwide. To avoid adverse reactions caused by the interactions between Western pharmaceuticals and CHM, consumers should be encouraged to communicate with their healthcare practitioners about any use of herbal products, and practitioners should consider initiating such discussions [38]. It is also recommended that appropriate training be provided to medical practitioners and pharmacist on basic knowledge of CHM and ways to access the relevant information (e.g. drug-herb interaction database), so that they can be aware of and avoid potential interactions between orthodox drugs and CHM. In addition, pharmacists should provide adequate and accurate safety information about herbal products if available [39,40]. Greater dialogue between TCM manufacturers, retailers and Western health professionals is required to develop effective safety measures for OTC users. Information regarding evidence-based drug–herb interaction studies and contradictions should be further developed and made widely available for clinicians and healthcare practitioners, pharmacists, nurses and other allied health practitioners through various methods (i.e., online database, textbooks, education services, training, etc.).

## 3. Challenges to the Efficacy of CHM

Although CHM has been used over thousands of years, there is still a lack of scientific evident to support its efficacy in many cases [21]. The multi-component and multi-target mechanism of CHM is inherently distinct from often the mono-substance and mono-target model of WM, so utilising the Western evaluation paradigm to investigate the efficacy of CHM is not adequate or efficient [16]. The difficulty of developing a research methodology that is suitable to study the complex nature of herbal formulae and reaching a stage where CHM produces products of assured efficacy are but two of the many challenges still facing CHM.

### 3.1. Non-Uniform Chemical Composition Leading to Inconsistent Therapeutic Effects

The inconsistent quality of the herbal material is likely to induce fluctuating levels of therapeutic effects, which represent one of the major concerns for their effectiveness. Various factors (e.g., resource, complex nature, environmental factors) can affect the chemical composition and content of active components in CHM, and that may be the reason for inconsistent therapeutic effects. For example, Kam et al. (2013) used *Punica granatum* L. (known as *Shiliupi* in CHM) as a case study and suggested that herbs collected from different origins can have significantly different levels of anti-oxidant effects. The polyphenolic content and the corresponding anti-oxidant capacities of the methanol extracts of pomegranate peel were compared among samples collected from Australia, China and the US. The results showed that *Punica granatum* L. from China generally had a significantly stronger total anti-oxidant capacity than that from Australia and the US, which was strongly correlated with the higher total phenolic and flavonoid contents [41]. In addition, a study from Dong et al. (2003) revealed that *Panax notoginseng* F.H.Chen (known as *Sanqi* in CHM) cultivated in the south-western parts of Wenshan during September to October presented the best quality in terms of the amount of the bioactive compounds such as saponin, flavonoid and polysaccharides [42]. Those bioactive compounds were reported to have a variety of bioactivities, such as anti-oxidant, anti-depressant, anxiolytic and anti-inflammatory activities [43,44]. Hence, these studies highlighted that the non-consistent chemical composition of CHM greatly affects biological activity, which raises a significant challenge for developing quality standardisation of the herbal materials and CHM products.

### 3.2. Multiple Clinical Endpoints

In TCM, herbal formula is usually designed according to TCM pattern diagnosis to improve the overall function of the body. From TCM’s point of view, a disease presents in a whole and dynamic way; that is, it involves interactions between many biological systems in the body and these interactions change as the disease improves or worsens. Thus, the ‘endpoint’ of TCM treatment is the pattern that reflects multi-system changes [45]. Benefits produced by herbal medication include not only the regulation of several crucial biological targets but also, more importantly, the modulation of other associated general changes that delay the healing process. Therefore, a herbal formula that is designed to treat a TCM pattern can be applied for several Western diseases [45]. For example, *Liu Wei Di Huang Wan* (LWDH) is a classic TCM formula used for the TCM pattern ‘*Yin* deficiency and tonify *Kidney*’. Nowadays, pharmacological studies have proven that this formula is effective for multiple diseases, including metabolic syndrome, diabetes and cardiovascular disease, based on long clinical experiences and pharmacological studies [1]. As there is no direct interpretation between TCM patterns and Western pathophysiology, elucidating the pathological mechanisms and translating TCM indications into Western clinical endpoints remains a great challenge.

### 3.3. Non-Standardised Ratio of Herb Ingredients

The dose of herbal ingredients is an essential part of a CHM formula. TCM practitioners may adjust the ratio of herbal ingredients in the formula according to the improvement or aggravation of the symptoms, in order to achieve an optimal therapeutic effect. That is, different proportions of herb ingredients in a formula may totally alter the therapeutic effects and lead to different clinical outcomes. For example, a greater proportion of *Salvia miltiorrhiza* Bge. (known as *Danshen* in CHM) is used in the Danshen-Sanqi formula for cardiovascular management. However, the dosage of *Danshen* should be reduced when the formula is used for haemostasis [46]. In TCM clinics, there is no standardised ratio for a formula, especially when it is prescribed as a decoction. The ratio is designed by a practitioner based on clinical experience or traditional knowledge. It is difficult to systematically review the ratios used in the formulae and explain their rationale. The standardised ratio in Chinese patent medicines for specific clinical indications may give a clue. However, due to inadequate intellectual protection for CHM products in China, several manufacturers have kept the ratio confidential for their patent products. The non-standardised proportion of herb ingredients in traditional herbal decoctions make it difficult for researchers to establish their efficacy, as the therapeutic effects may not be optimal if the dosage of ingredients is incorrect.

### 3.4. Synergy and Synergistic Mechanism

Most of the current research on CHM is on the single compounds isolated from individual herbs, and, so far, millions of compounds have been isolated from plants, characterised and screened for therapeutic activities. However, understanding the action for a single compound is far from fully understanding the therapeutic activity of the whole herb and herbal formulation. As CHM is constituted by multiple herbal ingredients and hundreds of chemical compounds, the therapeutic effects depend on the overall interactions among all the components [7]. It is believed that the interaction exerts synergistic effects in a well-constructed formula so that the optimal clinical outcome can be obtained, and the key step to unravel the efficacy of CHM is to understand its synergistic mechanism.

Synergy is an intrinsic part of the philosophy of CHM, and it is an essential concept for constructing a herbal formula [47]. The synergistic concept in CHM involves enhanced therapeutic effects, reduced toxicity, network-target actions and promoted bioavailability [48]. The synergy concept in CHM formulae is introduced as compatibility principles, including *Xiang Xu* (synergism), *Xiang Shi* (assistance), *Xiang Sha* (elimination), *Xiang Wei* (detoxification), *Xiang Wu* (antagonism) and *Xiang Fan* (rejection) [47]. These principles are well established and extensively applied in TCM clinics, but the scientific rationale is still lacking. With the increasing popularity of Western drug combinational therapy, mathematic and computational models to quantitatively determine synergy (such as mathematic equations), computer bioinformatics technology and systems biology have been developed. These models were then gradually applied to explore the synergy and synergistic mechanism on CHM formulae. However, these models cannot effectively reflect the concept and draw useful conclusions for a holistic insight into CHM [49]. Due to the limits of current methodologies and laboratory techniques, CHM still confronts great challenges in investigating synergy to fully elucidate its efficacy.

### 3.5. Low Quantity and Quality of Efficacy Studies

Currently, most CHM products have not subjected to extensive scientific efficacy research before marketing. Scientific research on CHM is mainly published in the Chinese language [7]. The majority of these studies, especially early studies, are compromised by methodological flaws. For example, a systematic review reported that more than 2000 clinical trials can be found for Xiao Yao San (a classic TCM formula used for insomnia for over 2000 years) published in Chinese databases, and only two have met the standard of double/single-blinded randomised controlled trials [50]. Since a herbal formula is designed to treat TCM patterns rather than modern diseases, with treatment according to ‘syndrome differentiation’, and the formula ingredients are usually tailored to each person’s condition, thus it is rather difficult to design proper methodology for both clinical trials and pre-clinical studies. There are a large number of animal studies published in Chinese that show the overall effect of a single herb or a formula, but an exploration of the mechanism at the cellular, molecular and protein/gene levels is still needed. Moreover, many CHM products have not been subjected to scientific investigation to support clinical indications and potential therapeutic benefit [20]. Therefore, high-quality and rigorously designed clinical trials together with mechanistic studies are needed to show the efficacy of CHM.

## 4. Conclusions

This review has summarised the current status of CHM and outlined the major challenges for the development and modernization of CHM. Generally, the management of safety and efficacy for CHM is still under development. Due to CHM’s unique philosophy, diagnosis and prescription, which are completely different from WM, the safety control and efficacy evaluation and development of CHM in a modernised scientific manner has faced great challenges. More effort is needed to minimize the safety risks and provide scientific proof and clinical evaluation for the continuous development of this ancient medical therapy.
